# Insulin-like growth factor 1 myocardial expression decreases in chronic alcohol consumption

**DOI:** 10.1186/2050-490X-1-3

**Published:** 2013-10-01

**Authors:** Francesc Borrisser-Pairó, Emilia Antúnez, Ester Tobías, Joaquim Fernández-Solà

**Affiliations:** Alcohol Research Unit. Hospital Clínic. Institut d’Investigacions Biomèdiques August Pi i Sunyer (IDIBAPS). Department of Medicine, University of Barcelona, Barcelona, Spain

**Keywords:** Alcohol, Myocardium, IGF-1

## Abstract

**Background:**

Alcoholic cardiomyopathy (CMP) is one of the major complications of chronic excessive alcohol consumption. The pathogenic mechanisms implicated are diverse, inducing functional and structural changes in the myocardium. Insulin-like Growth Factor 1 (IGF-1) plays an important role in modulating the cell cycle, and helps the differentiation and proliferation of cardiac tissue inhibiting apoptosis. Experimental studies have suggested the role of IGF-1 in alcohol-induced cardiac damage. The aim of the present study was to determine the effect of chronic alcohol consumption on IGF-1 myocardial expression and to compare this expression in cases of hypertension and other cardiac diseases.

**Methods:**

We studied heart samples from human organ donors: 10 healthy donors, 16 with hypertension, 23 with chronic alcohol consumption and 7 with other causes of cardiac disease. IGF-1 myocardial expression was evaluated with a specific immunohistochemistry assay using a semi-quantitative method.

**Results:**

A significant decrease in IGF-1 myocardial expression was observed comparing all the cases included with control donors. This decrease in IGF-1 myocardial expression was significantly lower in the group of donors with chronic alcohol consumption compared to controls. On group evaluation according to the presence of CMP, donors with chronic alcohol consumption without CMP presented significantly lower IGF-1 expression than controls, whereas donors with chronic alcohol consumption with CMP showed a downward trend without achieving significance.

**Conclusions:**

Chronic alcohol consumption significantly reduces IGF-1 myocardial expression. This decrease induced by alcohol is partially compensated in the presence of structural myocardial damage.

## Background

Although there is evidence that moderate alcohol consumption has some beneficial effects in health [1], chronic and excessive alcohol intake can lead to the development of dilated cardiomyopathy (CMP) and other cardiovascular diseases [2–5]. Almost 1 in 3 alcoholic misusers develop heart disease, whether alcoholic CMP, cardiac hypertrophy, arrhythmias or reduced myocardial contractility [6].

Alcoholic CMP is one of the major complications of chronic alcoholism [5], developing in a dose-dependent manner, independently of vitamin or nutritional deficits [7–9]. The cumulative lifetime dose of alcohol needed to develop alcoholic CMP has been assessed as 7 kg per kg of body weight of ethanol in men and 5 kg in women [10]. Alcoholic CMP is characterized by progressive structural and functional changes in the myocardium that remain subclinical for years until the appearance of heart failure or arrhythmia [10]. There is also a direct relationship between cardiac dysfunction and skeletal myopathy in patients with alcoholic CMP [11].

The physiopathological mechanisms implicated in alcohol-induced toxic damage to striated skeletal and cardiac muscle are multifactorial and are probably additive [12, 13], being apoptosis, oxidative damage and contractile protein disruption the most relevant [3, 12]. In previous studies in chronic alcoholic donors, we observed an increase in myocardial apoptosis to a similar degree as that caused by hypertension. This effect was more prominent in alcoholic donors with already developed heart damage [14]. In another study we observed that hearts from donors without alcoholic CMP presented over-expression of L-type calcium channels, a fact that increases Ca^2+^ entry into the cells and induces myocyte damage [9].

Recently, some authors described the potential effect that diverse myocyte growth factors such myostatin and IGF-1 may exert in regulating the development of alcohol-induced heart damage. Myostatin is a growth factor that, when up-regulated, inhibits the proliferation of muscle and cardiac myocytes, for this reason it is down-regulated during tissue development [15]. We have reported an increase in myostatin myocardium activity in subjects with CMP either of alcoholic or non alcoholic origin [16].

Insulin-like Growth Factor 1 (IGF-1) is a simple string of polypeptides with a structure similar to proinsulin. IGF-1 has short-term effects on the proliferation and differentiation of different cell types including cardiac myocytes. Its mitogenic activity is mediated by its binding to a IGF-1 receptor found on the cell surface [17, 18]. Growth hormone (GH) is linked to IGF-1 receptor by IGF-1 promoting growth and tissue differentiation [19, 20]. The direct effect of IGF-1 on cells is related to DNA synthesis, cell cycle gene modulation and induction of cell entry to the S phase. IGF-1 activation induces cell proliferation and increases protein synthesis, resulting in cell hypertrophy and promoting tissue protection. It also has an anti-apoptotic effect mediated by inhibition of Bax induction, caspase activation and DNA fragmentation [17, 21, 22]. Local myocardium activity of IGF-1 is most relevant than that produced elsewhere (i.e. in the liver). In fact, the bioavailability of exogenous administered IGF-1 is limited, because of the rapid clearance (half time < 10 min) of this peptide hormone from the circulation [23].

Some studies have evaluated the role of IGF-1 and GH in cardiac function and induction of myocyte damage after an ischemic heart attack. Both GH and IGF-1 have been shown to have a therapeutic effect on heart failure [19, 20]. A study done in alcohol-fed rats demonstrated a reduction in IGF-I mRNA content in liver and skeletal muscle, compared with pair-fed control rats [24]. An experimental study on IGF-1 in mice showed that over-expression of this growth hormone has a beneficial effect on the myocardial dysfunction caused by excessive consumption of alcohol [2]. No previous clinical investigations have studied the direct effect of alcohol on IGF-1 in humans.

Considering the previously noted pro-apoptotic effect of ethanol [14] as well as the up-regulation of myocyte myostatin, [16], and based on data from experimental studies on myocardial IGF-1 effect, we aimed to evaluate the potential role of IGF-1 in the development of alcohol-induced cardiac damage. Thus, we hypothesized that chronic alcohol consumption has an inhibitory effect on IGF-1 expression, and may consequently worsen the recovery of damaged myocardial tissue. For this purpose, we studied samples of human myocardium tissue from organ donors. Donors were classified into different groups according to alcohol consumption, the presence of CMP or the existence of hypertension or other causes of myocardial damage. The main objective in the present study was to evaluate myocardium IGF-1 expression in human organ donors and determine the effect of chronic alcohol consumption, the presence of CMP, hypertension or other causes of myocardial damage.

## Results

We included a total of 57 hearts samples from human donors collected at the Hospital Clínic Transplant Unit between January 2006 to December 2008. One sample which was not adequately cryopreserved was excluded. Therefore, a total of 56 heart samples were finally evaluated. Heart samples were divided into 4 groups as follows: (1) 10 healthy donors without evidence of alcohol consumption, hypertension or other causes of heart disease (control group), (2) 16 non-alcoholic hypertensive donors, (3) 23 donors with chronic alcohol consumption and (4) 7 non-alcoholic donors with other causes of cardiac disease. Table 1 shows the epidemiological and clinical data of donors according to these four groups. Remarkably there were no differences related to age and gender distribution between groups. Alcoholics presented a higher tobacco use compared to healthy non-alcoholic donors. None of these subjects presented criteria of caloric or protein malnutrition. As expected, donors with chronic alcohol consumption, hypertension and other cardiac diseases presented significant changes in echocardiography parameters and also in morphometrical parameters of myocardial hypertrophy compared to controls.

**Table 1:** **Epidemiologic and clinical data of the different groups of heart donors**

	Control donors (n=10)	Hypertensive donors (n=16)	Alcoholic donors (n=23)	Donors with other causes of cardiac disease (n=7)
Age (y; mean (SD))	52.3 (22.1)	62.1 (10.7)	54.3 (10.0)	59.1 (13.8)
Male/female ratio (n)	3:7	8:8	19:4	6:1
Daily alcohol intake (g; mean (SD))	0	10.7 (19.4)	148.2 (54.9)***	8.6 (22.8)
Lifetime dose of ethanol (kg ethanol/kg body weight; mean (SD))	0	0.54 (0.2)	15.5 (8.3)***	0.77 (0.2)
Active smokers [n (%)]	1 (10)	4 (25)	17 (74)***	2 (29)
Time from admission to donation (h; mean (SD))	30 (2)	30 (3)	33 (2)	30 (2)
NYHA function [n (%)]				
Class I	10 (100)	11 (68)	12 (51)	4 (57)
Class II	0	4 (26)	7 (30)	2 (29)
Classes III and IV	0	1 (6)	2 ( 9)	1 (14)
Cardiothoracic index (mean (SD))	0.47 (0.01)	0.54 (0.05)**	0.54 (0.05)**	0.58 (0.05)**
Electrocardiogram [abnormal cases; n (%)]	1 (10)	11 (69)*	10 (43)**	7 (100)*
Left ventricular ejection fraction (%; mean (SD))	61 (3)	45 (9)**	45 (7)**	41 (7)**
End-diastolic diameter (mm; mean (SD))	46.6 (2.2)	52.5 (5.1) **	58.4 (6.2) **	59.6 (6.4) **
End-systolic diameter (mm; mean (SD))	29.7 (2.9)	38.3 (3.8) **	43.8 (5.7) **	45.4 (6.2) **
Left ventricular mass (g/m^2^; mean (SD))	107 (6)	139 (10) **	143 (11) **	146 (13) **
Cell hypertrophy [n (%)]	0 (0)	11 (69) **	17 (74) **	7 (100) **
Nuclear hypertrophy [n (%)]	0 (0)	14 (88) **	19 (82) **	7 (100) **

An abnormal electrocardiogram is characterized by the presence of rhythm disturbances, conduction defects, signs of left ventricular hypertrophy, or abnormal repolarization.

* P < 0.05 compared to the other groups.

** P < 0 .01 compared with control donors.

*** P < 0.01 compared to the other groups.

To evaluate the effect of CMP on IGF-1 myocardial expression, these cases were further divided in 6 groups: (1) 10 healthy non-alcoholic donors, (2) 8 hypertensive non-alcoholic donors without CMP, (3) 8 hypertensive non-alcoholic donors with CMP, (4) 11 donors with chronic alcohol consumption without CMP, (5) 12 donors with chronic alcohol consumption with CMP and (6) 7 non-alcoholic donors with other causes of CMP (2 ischemic disease, 3 valve disease and 2 idiopathic CMP).

As expected, the cases (groups 2, 3, 4, 5 and 6) presented a higher cardiothoracic index, lower left ventricular ejection fraction and electrocardiogram abnormalities and showed a worse clinical class than controls (group 1). The consumption of daily alcohol intake (38.57 ± 12.10 vs. 43.94 ± 11.66, in g/day; p=0.676) and lifetime dose of ethanol (5.04 ± 2.54 vs. 3.50 ± 1.10 in Kg of ethanol/Kg body weight; p=0.598) and tobacco consumption (10.43 ± 2.82 vs. 10.30 ± 2.66 in cigarettes/day; p=0.892), was similar in the group of alcoholic donors when divided according to the presence of structural CMP.

### IGF-1 immunohistochemical myocardial expression

**Figure 1:**
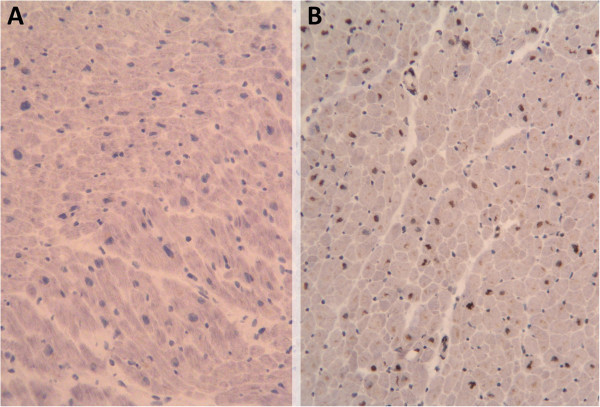
**Insulin-** **like growth factor 1** **(IGF-** **1)** **immunohistochemical expression in myocardium at 10 μm** **(magnification ×100).** **(A)** Negative control without IGF-1 antibody. **(B)** Positive control sample from a healthy donor with IGF-1 antibody.

**Figure 2:**
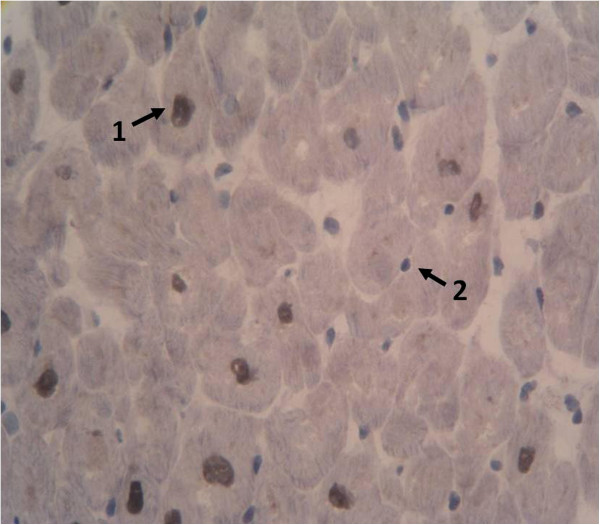
**Insulin-** **like growth factor 1** **(IGF-** **1)** **immunohistochemical expression in myocardium at 10 μm** **(magnification ×400).** **(1)** IGF-1 positive nuclear expression. **(2)** Negative nucleus without IGF-1 expression.

We first evaluated the presence of appropriate immunohistochemical IGF-1 myocardial expression. Thus, samples without IGF-1 antibody were observed to be uniformly negative only with hematoxylin staining, whilst samples with IGF-1 antibody showed clearly positive expression of IGF-1, with dark brown staining in the nucleus (Figure 1). In a magnified image (Figure 2) we can see further staining in nuclear expression of cardiac cells with some showing clear IGF-1 expression and other cells with absent IGF-1 expression.

**Figure 3:**
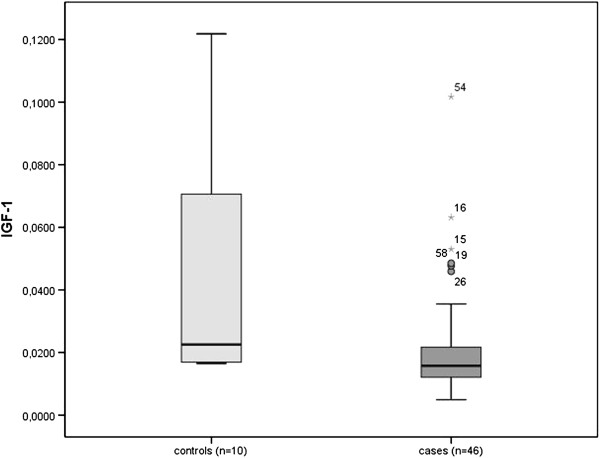
**Box-** **plot comparing healthy donors and cases with respect to Insulin-** **like growth factor 1** **(IGF-** **1)** **index expression** **(p=** **0.** **007).**

With respect to IGF-1 immunohistochemical expression, on division of the samples into healthy non-alcoholic donors (controls) and cases (the latter including hypertension, chronic alcohol consumption and other causes of cardiac disease), the IGF-1 expression index was 0.0439 ± 0.0127 versus 0.0219 ± 0.0026, respectively, with a significant decrease in IGF-1 myocardial expression in cases compared to controls (p=0.007) (Figure 3).

Then, we compared IGF-1 myocardial expression in healthy non-alcoholic donors used as controls with hypertensive donors, donors with chronic alcohol consumption and donors with other causes of cardiac disease separately (Table 2). Remarkably, we just found a significant decrease in IGF-1 myocardial expression on comparing donors with chronic alcohol consumption with controls (0.0206 ± 0.0042 vs. 0.0439 ± 0.0127, respectively, p=0.002). Hypertensive donors tended to express low IGF-1 myocardial expression compared to controls, albeit without achieving statistical significance (0.0250 ± 0.0043 vs. 0.0439 ± 0.0127, respectively, p=0.068).

**Table 2:** **Comparison of myocardial immunohistochemmical studies in IGF**-**1 expression between the different groups**

Number of samples used for immunohistochemical study: n=56	IGF-1 myocardial expression	p-value compared with control donors
Control donors (n=10)	0.0439 (0.0127)	
Hypertensive donors (n=16)	0.0250 (0.0043)	0.068
Hypertensive donors without CMP (n=8)	0.0264 (0.0074)	0.101
Hypertensive donors with CMP (n=8)	0.0237 (0.0052)	0.173
Chronic alcohol consumption donors (n=23)	0.0206 (0.0042)	0.002
Chronic alcohol consumption donors without CMP (n=11)	0.0132 (0.0012)	0.000
Chronic alcohol consumption donors with CMP (n=12)	0.0264 (0.0074)	0.069
Donors with other causes of cardiac disease (n=7)	0.0191 (0.0014)	0.161

Data are expressed as IGF-1 index expression (SD).

*IGF-1* Insulin-like Growth Factor.

*CMP* Cardiomyopathy.

In order to assess the influence of the presence of structural CMP in IGF-1 myocardial expression, we compared IGF-1 expression index in all cases and controls divided according to the presence or absence of structural CMP (n=29 in donors without CMP donors, and n=27 in donors with CMP). Thus, the IGF-1 myocardial expression in the two groups did not significantly differ (0.0274 ± 0.0053 in donors without CMP compared to 0.0241 ± 0.0037 in donors with CMP, p=0.749). We also evaluated the relationship between IGF-1 myocardial activity and the presence myocyte hypertrophy. Thus, myocyte IGF-1 activity was higher in donors without nuclear morphometric hypertrophy than in donors with nuclear hypertrophy (0.037 ± 0.009 vs 0.022 ± 0.0038, P= 0.020). Similarly, IGF-1 myocyte activity in donors without cell hypertrophy was significantly higher compared to their counterpart not affected of cell hypertrophy (0.034 ± 0.006 vs 0.020 ± 0.002, P= 0.048). IGF-1 myocardial activity was similar in alcoholics with higher tobacco use and alcoholics with non-tobacco use.

Finally, the IGF-1 myocardial expression of non-alcoholic healthy controls was separately compared with that of each group of cases according to the presence of structural CMP. Only the group of donors with chronic alcohol consumption non-affected of CMP showed significantly lower IGF-1 myocardial expression compared to non-alcoholic controls (0.0132 ± 0.0012 vs. 0.0439 ± 0.0127, respectively, p=0.000). Donors with chronic alcohol consumption with CMP tended to express lower IGF-1 myocardial expression, but without achieving significant differences compared to non-alcoholic controls (0.0264 ± 0.0074 vs. 0.0439 ± 0.0127, respectively, p=0.069). No differences were obtained on comparing the controls with the other groups of cases (Table 2). When we compare donors with chronic alcohol consumption with CMP versus chronic alcohol consumption without CMP no significant differences were found (p=0.169).

Lastly, we performed a regression analysis between parameters of ethanol consumption and IGF-1 myocardial expression, but did not find a significant correlation between them (R^2^= 0.0939 for mean daily alcohol consumption and R^2^ = 0.0759 for lifetime cumulative dose of alcohol and IGF-1 myocardial expression, respectively).

## Discussion

This study evaluates IGF-1 expression in myocardial tissue in human donors with chronic alcohol consumption in comparison to healthy donors and also with donors with hypertension or other cardiac diseases using an immunohistochemical IGF-1-specific assay. We attempted to assess the effect that chronic alcohol consumption may cause in IGF-1 myocardial expression in comparison to non-alcoholic controls and other different groups of donors and also evaluate the influence of the presence of structural CMP.

The main result achieved is the evidence that chronic alcohol consumption significantly reduces IGF-1 myocardial expression compared to non-alcoholic healthy controls. Otherwise, we did not observe significant differences in IGF-1 myocardial expression in the other groups evaluated, although hypertensive donors showed a non-significant downward trend. Remarkably, IGF-1 myocardial expression was lower in donors with myocardial hypertrophy compared to those without hypertrophy. This finding probably is related to a negative counter-regulation between myocyte IGF-1 activity and cell hypertrophy, corroborating the influence of this factor on myocardial hypertrophy. Finally, on evaluating the influence of the presence of structural CMP on IGF-1 myocardial expression we did not find significant differences between groups with structural CMP and those without. This IGF-1 decreased myocardial expression in alcoholic donors was clearly significant in those without CMP thereby excluding an effect of structural CMP on a decrease in IGF-1 myocardial expression. In fact, the presence of structural CMP in donors with chronic alcohol consumption non significantly increased IGF-1 myocardial expression with respect to donors without structural CMP, a fact that may be compensatory of the inflicted cardiac toxic damage. We also observed a downward trend in IGF-1 myocardial expression in the other groups of donors with hypertension and other cardiac diseases, although without achieving significant differences compared to controls.

Indeed, IGF-1 is a trophic factor with an important role and short-term effects on the proliferation and differentiation of different cell types including heart myocytes, IGF-1 increases cardiac DNA and protein synthesis and reduces protein degradation [17]. After myocardial infarction IGF-1 improves cardiac function by stimulating contractility and promoting tissue remodeling, a decrease in IGF-1 expression affects myocardial function and myocyte regeneration [17, 18].

The potential mechanism to explain this alcohol-mediated decrease in IGF-1 myocardial expression may be the decrease in protein synthesis and translation initiation that alcohol induces in the myocardium [25]. This effect causes a impairment in the availability and effectiveness of various anabolic hormones including IGF-1 [13] and myostatin [16]. These results are in concordance to those observed in experimental studies in mice in which alcohol-fed rats showed a reduction in the IGF-1 mRNA content in liver and skeletal muscle compared with pair-fed control rats [24]. In fact, it has been suggested that IGF-1 expression protects the myocardium tissue reducing apoptosis and increasing myocardial proliferation [17]. For this reason, an alcohol-induced IGF-1 myocardial decrease in expression is partly compensated in the presence of cardiac damage.

In the present series, alcohol donors consumed higher tobacco dose than non alcoholic donors. However, In the multivariate analysis we did not identify tobacco as a variable influencing myocardial IGF-1 expression. In addition, no biological effect between tobacco consumption and IGF-1 activity or expression has been reported up to now [26, 27].

In the present study we did not obtain a significant correlation between alcohol consumption parameters and IGF-1 myocardial expression. This may be due to the fact that ethanol consumption in the donors studied was not high but rather slight or moderate. In addition, it is clear that ethanol has diverse pathogenic effects on the myocardium and the global noxious effect may be a sum of diverse implicated mechanisms such as disturbance in calcium transients, induction of apoptosis, an increase in myostatin expression or induction of oxidative damage [8, 14, 16].

With respect to the hypertensive donors included in this study, they showed a downward trend of IGF-1 myocardial expression compared to healthy donors. This is in concordance to what has been observed in previous studies, in which low levels of IGF-1 were found to be associated with hypertension in subjects without other cardiovascular diseases [28].

One limitation of the present study was that it evaluated IGF-1 myocardial expression in a medium-size group of human donors, without considering other pathogenic mechanisms that may also be potentially implicated in alcohol-induced cardiac damage. The present study does not include data in IGF signaling cascade or complementary *in vitro* studies. The degree of daily and lifetime alcohol consumption in the group of donors with alcohol consumption was only moderate, thus the effect of high-dose ethanol intake may not have been considered in the present study. IGF-1 expression was only limited to cardiac immunohistochemical without IGF-1 receptor or intracellular IGF-1 signaling.

## Conclusions

We observed a significant decrease in IGF-1 myocardial expression in alcoholic donors without structural CMP compared to controls. This effect was not observed in donors with hypertension or other cause of cardiac disease. Since IGF-1 promotes cardiac growth, improves cardiac contractility, cardiac output, stroke volume, and ejection fraction, it has been suggested that IGF-1 has therapeutic potential [17, 22]. As previously reported in an experimental study in mice, over-expression of IGF-1 has a beneficial effect on the myocardial dysfunction caused by excessive consumption of alcohol [2]. For this reason, a potential therapeutic use of IGF-1 could be suggested in patients with chronic alcohol consumption to avoid progression to CMP.

## Methods

### Selection of patients and controls

In the transplant unit at the Hospital Clínic subjects with brain death due to trauma or cerebrovascular causes are routinely evaluated for possible transplantation. Of these donors under the age of 70 years, hearts which were not suitable for transplantation were consecutively separated and classified into 4 groups: (1) control hearts from healthy non-alcoholic people who were not eligible for implantation because of a lack of matched receptor or size inadequacy, (2) hypertensive non-alcoholic donors, (3) donors with chronic alcohol consumption (≥60 g/day for over 10 years), (4) non-alcoholic donors with other causes of cardiac disease (ischemic, valve or idiopathic).

All patients were white Caucasians of Spanish descent, who lived with their families in or around Barcelona and none was indigent. Some of these subjects had been included in previous studies on heart antioxidant status [8], cardiac apoptosis [14] and myostatin activity [16].

All cases had been admitted to the intensive care unit and ventilator and hemodynamic parameters had been appropriately maintained at normal values throughout hospitalization (PaO^2^ >60 mm Hg, systolic blood pressure >100 mm Hg, and arterial pH within the normal range). None of the patients required in-hospital cardiopulmonary resuscitation maneuvers. Because all patients were maintained in similar conditions of glucose homeostasis, we consider that insulin do not play a significant role in the final measured myocardial IGF-1 activity.

The study protocol was approved by the Ethics Committee of the Hospital Clínic and informed consent was requested from the families of the donors concerning the use of myocardium tissue for this research protocol study. The authors of this manuscript have certified that they comply with the statement on ethics from the HEART Group [29].

### Clinical and laboratory evaluation

A detailed history of ethanol intake was retrospectively obtained by consultation with family members using a structured questionnaire (“time-line follow-back method”) [30], as previously reported [10, 11]. Duration of ethanol intake was calculated in each group as the total cumulated period of alcohol consumption in years, either recent or previous. The body mass index (BMI) was determined as the actual body weight relative to the square of the body height (kg ⁄m^2^). Patients were considered to have caloric malnutrition if the BMI was <17 kg ⁄m^2^. Protein malnutrition was assessed by the following parameters obtained at hospital admission: hemoglobin, lymphocyte count, total protein, retinol-binding protein, prealbumin, and albumin.

### Cardiac studies

Past and present signs and symptoms of heart failure were evaluated on consultation of medical records and with family members of the donors, and the New York Heart Association (NYHA) functional class was determined according to the Goldman activity scale [31]. Chest X-ray with measurement of cardiothoracic index and conventional electrocardiography were performed in all cases. A cardiothoracic index greater than 0.48 was observed in 27 patients compared to none of the controls. In these donors with enlarged cardiothoracic index, bidimensional echocardiography was performed (Hewlet Packard Sonos 2500; Hewlet Packard, Andover, MA). End-diastolic and end-systolic diameters, the shortening fraction, left ventricular (LV) mass, and the ejection fraction (EF) were measured according to the standards of the American Society of Echocardiography [32]. Cardiomyopathy (CMP) was defined as the presence of LVEF <50% and LV enlargement. We observed a good correlation between the cardiothoracic index and the LV end-diastolic diameter (r=0.68, p<0.01). The personnel performing and evaluating these tests had no knowledge of the alcoholic history of the patients.

### Myocardium histological studies

A 3 cm distal sample of the LV apex was surgically excised avoiding damaged areas (total weight of 4 to 5 grams) at the time the donor was under cold perfusion. The specimen was cut into fragments, and one of these was processed for further histological analysis.

### Immunohistochemistry

Myocardial samples were preserved at −80°C. Immunohistochemical processing required cutting of samples to 10 μM using cryotome and fixation on glass slides. The slices were kept frozen (−80°C) until the time of use. Slices were fixed with acetone for 10 minutes and then proceed to warming in the oven at 95°C for 30 minutes with citrate buffer (2.94 g Tri-sodium citrate + 1000 ml H_2_O + 0.5 ml Tween 20 at pH 6 with HCl 1N) and let cool for 20 minutes at room temperature. Slices were cleaned with PBS and blocked with H_2_O_2_ (1%) for 15 minutes and were thereafter cleaned again with PBS.

Immunohistochemical analysis for IGF-1 expression was chosen because myocardial IGF-1 expression was considered to be the most relevant parameter in the physiopathology of myocardial IGF-1 effects, as previously reported in other studies [33, 34]. Immunohistochemical detection of IGF-1 expression was evaluated using a commercial kit of a polyclonal rabbit antibody (IGF-1 antibody, ab9572, Abcam, Cambridge, UK). The antibody is a recombinant immunogen (human Insulin-like Growth Factor-1). The antibody dilution used was 4 μg/ml in a solution of blocking serum 1.5% in PBS and was incubated overnight at 4°C. In each sample we also performed a negative control processed without primary antibody. Detection was performed by the compatible secondary antibody (rabbit ABC Staining System, sc-2018, Santa Cruz Biotechnology Inc., Santa Cruz, CA) linked to peroxidase. A last cleaning with H_2_O was performed and slices were stained with Gill’s hematoxylin 2 for 10 minutes and cleaned with H_2_O for 5 minutes. Finally, slides were prepared with an aqueous mounting agent (Aquatex, Darmstadt, Germany).

### Microscopic evaluation

Myocardial cell and nuclear hypertrophy was evaluated by histological morphometry, as previously reported [11]. IGF-1 myocardial expression was evaluated with immunohistochemical methods using optical microscopy at ×200 magnification with a semiquantitative approach, counting the percentage of positive cells with respect to total evaluated myocardial cells. We measured the positive IFG-1 expression in both the nucleus and cytoplasm of the myocytes. Six areas of each sample were evaluated, including 200 to 600 cells per field. A minimum of 1,200 cells per sample were counted. IGF-1 expression index was calculated according to the ratio between positive-stained myocytes divided by negative-stained myocytes. Results from control donors were compared to those of hypertensive, chronic alcohol consumers and donors with other causes of cardiac disease. In the three last groups we also analyzed the effect of the presence of structural CMP on IGF-1 myocardial expression.

### Statistical analysis

The data were analyzed using SPSS-PC 18.0 statistical software (SPSS, Chicago, IL). Firstly, descriptive statistics were calculated and tested for normality (Kolmogorov-Smirnov). Although the groups follow a normal distribution due to the small sample size, we used a nonparametric test (Mann–Whitney) to assess the presence of significant differences between the parameters studied. A significance level lower than 0.05 was used.

## Abbreviations

CMP: Cardiomyopathy
EF: Ejection fraction
GH: Growth hormone
IGF-1: Insulin-like growth factor-1
LV: Left ventricular.
